# 

**Published:** 1888-05

**Authors:** 


### AUSTIN DISTRICT MEDICAL SOCIETY.

The following circular has been issued by the Secretary of the Austin District Medical Society.

AUSTIN, TEXAS, May 14, 1888.

The fourth quarterly meeting of the Austin District Medical Society will be held in the city of Austin on Thursday, the 14th of June, *prox.* You are respectfully invited to be present.

This Society, though less than one year old, has attained a remarkable growth and strength, and now numbers on its roll of active members not less than fifty of the best known and ablest physicians in the district. Its meetings have been interesting, instructive and pleasant; and the amount of scientific work already accomplished is a matter of congratulation. An advantage possessed by a district society over the large and more or less unwieldy meetings of State Associations, is that all have opportunity to be heard, to take part in the discussion of papers presented, or to read papers; and the meetings are more of the nature of social or informal discussions of topics of interest to all practitioners—the complicated machinery of semi-political meetings being avoided. Nothing disparaging to the State Association is intended by thus emphasizing the District Society; both are working for the same end, viz: the elevation of the standard of medicine in Texas, thereby ennobling the profession and benefiting mankind.

The programme for this meeting is attractive, and embraces, in part, the following papers:

"The Pathology and Treatment of Endo-Cervical Inflammation;" by Dr. J. W. McLaughlin. Discussion will be opened by Dr. B. E. Hadra.

"Chloroform in Labor;" by Dr. J. W. Hamilton, of Webberville. Discussion by Dr. H. H. Thorpe, of Liberty Hill.

"Hemorrhoids, Fissure and Fistula in Ano, and their Treatment;" by Dr. T. J. Bennett. Discussion opened by Dr. L. C. Johnson, of Gidding.

In addition to the above, Dr. G. B. Underhill, of New Braunfels, and Dr. Sam Cunningham, of Elgin, have promised papers.

Dr. A. N. Denton will continue the subject of last meeting, viz: "The Management of the Insane in Texas."

Dr. F. R. Martin, of Kyle, will report from his practice three interesting cases of poisoning—one with what is known as Buckeye, one with coal oil, and the other with Sulphate of Copper combined with strychnia.

Papers and reports of interesting cases are always in order and invited.

Every reputable graduate from a recognized medical school is entitled to membership, and is urged to identify himself with the Society, in order that the public may know who are not entitled to confidence and recognition, who are quacks, and who are the live and progressive men of the district.

The Society will be called to order at 10 o'clock a. m. There will be an afternoon and a night session.

THOS. D. WOOTEN, Pres't.

T. J. BENNETT, Sec'y.

Married.—At Schulenburg, Texas, April 23, 1888, Dr. Isaac E. Clarke to Miss Ella Woolston, all of Schulenburg. The bridal couple visited Galveston during the meeting, and were recipients of many congratulations.

Married.—At Wichita Falls, April 30, 1888, Vice-President Texas State Medical Association, Dr. O. Eastland, to Miss — Jalonick, of that city. Our hearty congratulations are extended the handsome and accomplished doctor, and also to his happy young bride. They visited Denver and the Yellowstone for a bridal trip.

---

North Texas Medical Association.—The North Texxs Medical Association will hold its regular semi-annual meeting in the city of McKinney on June 12, 1888, and continue in session three days.

